# The Attitudes of Polish Women towards Breastfeeding Based on the Iowa Infant Feeding Attitude Scale (IIFAS)

**DOI:** 10.3390/nu13124338

**Published:** 2021-11-30

**Authors:** Agnieszka Bień, Bożena Kulesza-Brończyk, Monika Przestrzelska, Grażyna Iwanowicz-Palus, Dorota Ćwiek

**Affiliations:** 1 Chair of Obstetrics Development, Faculty of Health Sciences, Medical University of Lublin, 4-6 Staszica St., 20-081 Lublin, Poland; grazyna.iwanowicz-palus@umlub.pl; 2 Department of Obstetrics, Gynaecology and Maternity Care, Faculty of Health Sciences, Medical University of Bialystok, 1 Jana Kilińskiego St., 15-089 Bialystok, Poland; bozena.kulesza-bronczyk@umb.edu.pl; 3 Department of Nursing and Obstetrics, Division of Midwifery and Gynaecological Nursing, Faculty of Health Sciences, Wroclaw Medical University, 5 Bartla St., 51-618 Wroclaw, Poland; monika.przestrzelska@umw.edu.pl; 4 Department of Obstetrics and Pathology of Pregnancy, Faculty of Health Sciences, Pomeranian Medical University, 48 Żołnierska St., 71-210 Szczecin, Poland; dorota.cwiek@pum.edu.pl

**Keywords:** attitude, breastfeeding, Iowa Infant Feeding Attitude Scale, validity, Poland

## Abstract

Background: The Iowa Infant Feeding Attitude Scale (IIFAS), which is used for the assessment of attitudes towards breastfeeding, has been found to be reliable and valid in a number of countries, but has not yet been psychometrically tested in Polish women. The purpose of the study was to report on the cultural adaptation of the IIFAS to Polish settings and on its validation, to evaluate the breastfeeding attitudes in Polish women who recently gave birth, and to identify the determinants of these attitudes. Methods: The study was performed in a group of 401 women in their first postpartum days. Results: Cronbach’s α for the scale was 0.725. Discriminative power coefficients of all questionnaire items were higher than 0.2. Subscales were strongly correlated with the total score, with a correlation coefficient of 0.803 for the “favorable toward breastfeeding” subscale (*p* < 0.001), and 0.803 for the “favorable toward formula feeding” subscale (*p* < 0.05). For the item “A mother who occasionally drinks alcohol should not breastfeed her baby”, the factor loading did not reach the criterion value, and so the item was not included in further analyses. The mean IIFAS score was 63.12 (±7.34). Conclusions: The Polish version of the IIFAS is a reliable and appropriate measure of women’s attitudes towards infant feeding in Polish settings, with acceptable psychometric properties and construct validity.

## 1. Introduction

According to the WHO, the American Academy of Pediatrics, and the European Society for Pediatric Gastroenterology, Hepatology and Nutrition (ESPGHAN), breastfeeding is the optimal way of feeding infants and young children. It is also good for mothers, families, and society at large, with a number of specific health, environmental, and economic benefits [1,2,3]. Compared to formula feeding, breastfeeding reduces infant perinatal mortality and prevents a number of both childhood and adult diseases. Increased use of breastfeeding could prevent the deaths of 823,000 children under 5 and 20,000 breast cancer deaths annually [4].

Though the benefits of breastfeeding are well documented, the time of initiation and the duration of breastfeeding vary greatly around the world [5]. Efforts to promote breastfeeding are being undertaken on the global, national, or even individual level, and involve raising awareness of breastfeeding and motivating women to initiate it. The initiation and duration of breastfeeding depend, among other factors, on demographic and psychosocial characteristics, views of family members and health professionals, the health care system and social environment, and national health policy [6,7]. Other factors include the support received, as well as the mother’s knowledge, willingness, attitude, and decision about the way of feeding her newborn baby. Views and attitudes towards breastfeeding are significant to infant nutrition and are extensively studied [8,9,10,11,12,13,14].

WHO European Region Member States, which include Poland, have the lowest exclusive breastfeeding rates in infants aged 6 months (<25%) [3,15]. Global Breastfeeding Collective guidelines aim to promote strategies encouraging breastfeeding worldwide and to increase the rate of infants who are exclusively breastfed for the first 6 months up to 50% by 2025 [16]. Data on Poland in the report are limited due to insufficient monitoring in the country [17].

An understanding of mothers’ knowledge on and attitudes towards breastfeeding plays a role in the development and implementation of public health policies, as well as in the evaluation of interventions aiming to increase the breastfeeding rate. This is why up-to-date, reliable instruments are needed to assess breastfeeding knowledge and attitudes, and a more complete understanding of factors that affect women’s decisions and attitudes with regard to breastfeeding seems necessary. A study conducted by Lis-Kuberka and Orczyk-Pawiłowicz (2021) among Polish women showed that the women had a moderate level of knowledge about the short- and long-term benefits of breastfeeding [18]. On the other hand, a study by Baranowska et al. (2019) conducted among medical personnel providing care to women in the perinatal period demonstrated that they had a low level of knowledge about the benefits of breastfeeding beyond twelve months [19]. As there have yet to be any Polish studies on women’s attitudes towards breastfeeding using standardized instruments, which would ensure the comparability of findings with those reported by researchers in other countries, an investigation on the subject is indeed warranted. One of such instruments is the Iowa Infant Feeding Attitude Scale (IIFAS) developed by De la Mora and Russell. The scale is used to assess the attitudes of women towards feeding infants, and to predict the feeding choice (breastfeeding, combined, formula) and the duration of breastfeeding in various populations [20]. The IIFAS has been found reliable and valid in a number of countries, but is yet to be psychometrically tested in Polish women.

### Purpose of the Study

The purpose of the study was threefold: to report on the cultural adaptation of the scale to Polish settings and its validation; to evaluate the breastfeeding attitudes in Polish women who recently gave birth; and to identify the determinants of these attitudes.

## 2. Materials and Methods

### 2.1. Assessments

The study used a diagnostic survey with questionnaires. The instruments used were the Iowa Infant Feeding Attitudes Scale (IIFAS) and a standardized interview questionnaire comprising questions on the participants’ characteristics (age, residence, education, relationship status, self-assessed socio-economic status, work before pregnancy, return to work after the end of maternity leave, mode of delivery, parity, way of feeding the previous baby, planned way of feeding now).

The IIFAS aims to evaluate women’s attitudes towards infant feeding and predict the chosen feeding method and duration of breastfeeding. It comprises 17 statements rated on a 5-item Likert scale, from 1 (strongly disagree) to 5 (strongly agree). Points in questions: 1, 2, 4, 6, 8, 10, 11, 14, 17 should be reversed (i.e., 1 = 5, 2 = 4, 4 = 2, 5 = 1), and the scores for each item then summed together. Nine items have wording favorable to breastfeeding, and the remaining ones favorable to formula feeding. The total IIFAS score ranges between 17 and 85, with higher scores indicating a positive attitude towards breastfeeding. Totals can be classified as follows: (1) positive attitude towards breastfeeding (IIFAS scores of 70–85), (2) neutral attitude (IIFAS scores of 49–69), and (3) positive attitude towards formula feeding (IIFAS scores of 17–48). Cronbach’s α internal consistency coefficient ranges between 0.85 and 0.86 [20].

### 2.2. Translation Procedures

The use of the original survey in the present study was officially approved by its author, Dr. A. De la Mora [20]. The questionnaire was translated from English into Polish by two independent translators, who were native speakers of Polish fluent in English. The resulting Polish translations were compared and checked for differences, and a single version was developed on their basis. This version was reviewed by specialists in infant feeding (a pediatrician and numerous midwives) who identified any cases of imperfect wording or phrasing in the translated version and proposed alternatives. This resulted in a version ensuring meaning equivalence for all items. Subsequently, the Polish questionnaire underwent back-translation into English, again performed by two translators other than those involved in the first step of the process. Following approval by the author of the original, the Polish version of the questionnaire (IIFAS-Pol) was assessed for the basic psychometric properties—reliability and validity. A pilot study was performed in a group of 30 postpartum women to verify comprehension of the questionnaire items.

### 2.3. Study Groups

The present study was performed between February 2020 and March 2021 in three stages: the first stage was conducted among postpartum women 2–4 days after delivery; the second—6–7 weeks after delivery; and the third—6 months after delivery. Stages 2 and 3 of the research were performed among the same respondents who were qualified to participate in the 1st stage.

The 1st stage was conducted among women who gave birth 2–4 days previously in obstetric wards of hospitals in 4 Polish provinces: Lublin, Podlasie, Western Pomerania, and Lower Silesia. Two are located in eastern Poland (Cardinal Stanisław Wyszyński Regional Specialist Hospital in Lublin and the Białystok University Hospital), and two in western Poland (the Pomeranian Medical University in Szczecin and Jan Mikulicz-Radecki University Hospital in Wrocław). Inclusion criteria were: 2–4 days postpartum, delivery at term (between 38th and 41st gestational week), hospital delivery, singleton pregnancy, and newborns placed with the mother after delivery. Exclusion criteria were: delivery before the 38th gestational week, clinical condition of the newborn necessitating separation from the mother, diagnosis of birth defects in the newborn, poor health of the mother (based on her medical records), or the mother’s psychological condition preventing breastfeeding.

The 2nd stage was performed in a group of 289 women from the 1st stage, 6–7 weeks postpartum, using follow-up questionnaires. Data on the women’s attitudes were collected by their midwives during the patronage visit.

The 3rd stage of the research was conducted among 206 women, 6 months postpartum. The data were collected remotely: each respondent received a paper questionnaire during the patronage visit and was asked to complete and return it 6 months after delivery.

**Figure 1:**
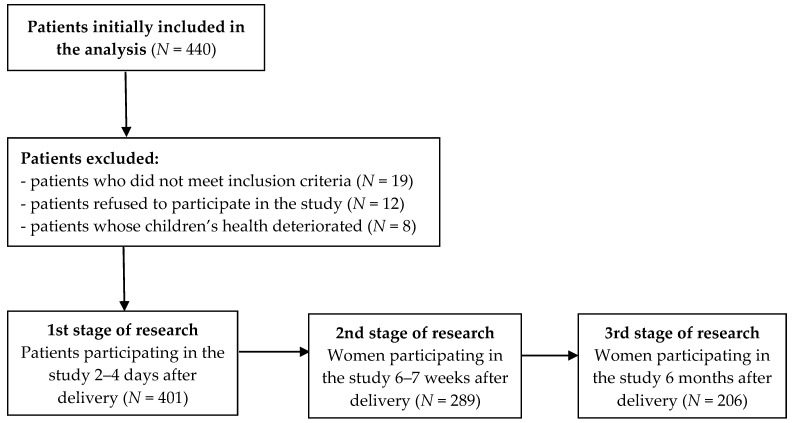
Flowchart of the recruitment process of the patients.

In part one of the study, 440 surveys were distributed; 401 correctly completed questionnaires were returned; and 39 patients were not included in the study for the following reasons: 19 patients failed to meet the inclusion criteria; 12 declined to participate; and 8 children required specialist treatment and separation from the mother due to a deterioration of their health (Figure 1). The survey response rate was 91.14%.

The study was approved by the Lublin Medical University Bioethics Committee (approval no. KE-0254/340/2019). Respondents were informed that participation was voluntary, and that study results were anonymous and to be used exclusively for research purposes.

### 2.4. Statistical Analysis

Cronbach’s α was used to assess the reliability of the scale measured by its internal consistency. Sampling adequacy was verified using the Kaiser–Mayer–Olkin test. Theoretical validity was assessed using exploratory factor analysis by the principal component method, applying a direct Oblimin rotation and Kaiser normalization. Instrument reliability was measured by the discriminative power of items constituting the identified dimensions. Subscale correlations with the total score were determined using Pearson’s r correlation coefficient. The impact of selected socio-demographic factors on women’s attitudes towards breastfeeding was evaluated using Student’s *t*-test and single-factor analysis of variance (ANOVA). ANOVA with repeated measures was used to compare three group means, where the participants are homogeneous in each group. The Kolmogorov–Smirnov test was applied to test for a normal distribution. Differences or correlations at *p* < 0.05 were considered statistically significant. Statistical analyses were performed using the IBM SPSS Statistics v. 26 software (Tibco Software Inc., Palo Alto, CA, USA).

## 3. Results

The study included 401 women. All women included in the study were white, aged between 18 and 43 years, and spoke and understood Polish. The mean age was 29.90 years (SD ± 4.95). Most patients lived in province capitals (62.3%), had completed higher education (62.6%), were married or in a steady relationship (84.8%), and assessed their socio-economic status as average (56.9%). Most of the women worked professionally before the pregnancy (82.0%) and intended to return to work after their maternity leave (80.5%). Most respondents had a vaginal delivery (54.1%), had given birth for the first time (48.6%), planned to breastfeed their baby (80.8%), and breastfed their previous children, if any (64.1%)—Table 1.

The internal consistency and reliability analyses for the Polish IIFAS version are shown in Table 2. The validity of the IIFAS questionnaire was tested by factor analysis. Factors were extracted from the correlation matrix by principal component analysis (PCA). Sampling adequacy measured by the Kaiser–Mayer–Olkin test was 0.671. This measure compares partial correlation coefficients with bivariate correlation coefficients. It takes on a value from 0 to 1. The value should not be lower than 0.5, as in a case such as this, the expected reduction would be small. Bartlett’s sphericity test was statistically significant (*p* < 0.001, chi-squared = 1289.327), showing adequate sampling. Factors were rotated, i.e., subjected to linear transformation. Rotation allows for a situation in which each variable has a high factor loading only on one factor, and every factor has at least several high loadings. This allows for obtaining a set of factors that is easier to interpret as compared to the primary factors produced without rotation. The Equamax rotation was applied to minimize the number of variables with high factor loadings and the number of factors required to explain the variables in the analysis.

Based on the theoretical assumptions of the questionnaire, a two-factor solution was enforced. Each factor was interpreted based on the primary variables with high factor loadings. In the present study, significant factor loadings were >0.3 [21]. For the item “A mother who occasionally drinks alcohol should not breastfeed her baby”, the factor loading did not reach the criterion value, and so the item was not included in further analyses.

**Figure 2:**
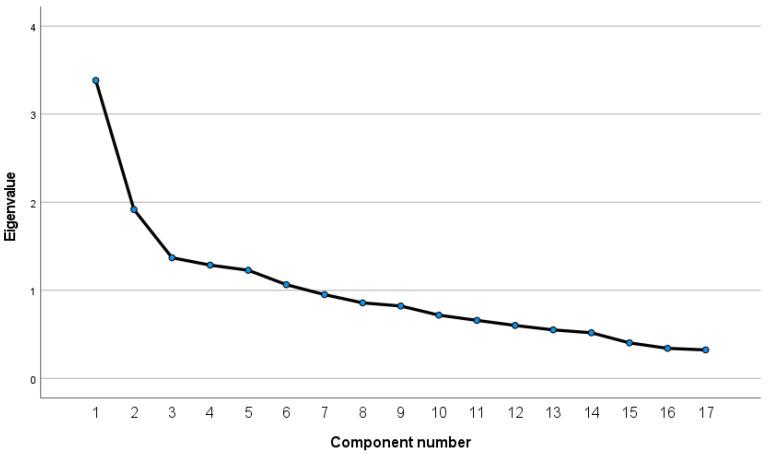
Scree plot of the 17-item IIFAS.

The two-factor solution explained 31.18% of variance. Factor 1 had an eigenvalue of 3.383 and accounted for 19.90% of variance. The factor was associated with a positive attitude towards breastfeeding and included 8 items. Factor 2 also included 8 items and accounted for 11.28% of variance. Its eigenvalue was 1.918. It was associated with a positive attitude towards formula feeding. The visual assessment of the two-factor solution was also made on the basis of the scree plot (Figure 2). The scree plot helps to determine the number of factors. The scree plot shows that the curve essentially flattens out after the second factor.

The reliability of the 17-item scale, measured by Cronbach’s α, was 0.678. The removal of item 17 (A mother who occasionally drinks alcohol should not breastfeed her baby) improved the scale’s reliability. Cronbach’s α for the 16-item scale was 0.725. The Cronbach’s α for each item deleted remained above 0.71, demonstrating the reliable use of the IIFAS in postpartum women in Poland. Discriminative power coefficients of all questionnaire items were higher than 0.2, and ranged from 0.201 to 0.492. For an exploratory study, 0.20 is an acceptable value for the item-total correlation [22]. Subscales were strongly correlated with the total score, with a correlation coefficient of 0.803 for the “favorable toward breastfeeding” subscale (*p* < 0.001), and 0.803 for the “favorable toward formula feeding” subscale (*p* < 0.05).

Scale reliability was also calculated for two groups: women who plan breastfeeding and women who plan formula or combined feeding. In the former group, scale reliability was 0.693, and in the latter 0.696. An additional analysis was performed in women who already had children, with the group broken down into those who had breastfed and those who had used formula or combined feeding. In the former subgroup, reliability was 0.603, and in the latter 0.801.

Table 3 shows mean scores for women’s attitudes towards breastfeeding in the first 2–4 days postpartum, after 2 months, and after 6 months. Our analysis showed no change in the women’s attitudes towards breastfeeding over time (*p* > 0.05).

A positive, weak correlation between women’s age and attitude towards breastfeeding (*p* < 0.031) was found in our study. Higher IIFAS results, indicating a more positive attitude towards breastfeeding, was observed in women living in the voivodeship capital city (*p* = 0.041), who completed higher education (*p* = 0.030), were married/in a relationship (*p* < 0.001), assessing their socioeconomic conditions as very good (*p* = 0.032), those who worked before pregnancy (*p* < 0.001), those who planned to breastfeed their baby in the first days after delivery (*p* < 0.001), and those who had breastfed their previous baby (*p* = 0.010). Parity and mode of delivery had no impact on breastfeeding attitudes (*p* > 0.05)—Table 4.

## 4. Discussion

Since 1997, there were no nationwide epidemiological studies in Poland focusing on breastfeeding. Only in 2014, for the first time in years, did Statistics Poland publish data on breastfeeding, showing that 92% of women start breastfeeding right after delivery, while 42% continue it in months 2–6 (the data were not collected in accordance with the WHO guidelines, but with the Polish children’s immunization program) [23]. Though obstetric-neonatal wards in Polish hospitals are required to protect, promote, and support breastfeeding, official data on the topic are still not being collected [24]. There are currently no guidelines allowing for standardization of the data collection system, and the methods of data collection differ among countries. Most countries gather information on breastfeeding rates, but unfortunately these data are inconsistent, sometimes inaccurate, and often incomplete. Data collection on breastfeeding occurs in a variety of formats, which can broadly be grouped into surveys of breastfeeding, and epidemiological studies and trials [25].

To understand the infant feeding attitudes of Polish mothers, we set out to develop and validate a Polish version of the feeding attitude questionnaire, and applied it to identify the determinants of these attitudes in accordance with the international standards described in the methodology section of the present paper. In our study, we used the IIFAS designed by Dr Arlene De la Mora [20]. The IIFAS questionnaire has been adapted and verified in a number of countries, showing good predictive validity and excellent internal consistency, with a Cronbach’s α ranging between 0.79 and 0.86 [8,9,14,20,26].

We analyzed internal consistency based on correctly completed questionnaires in part one of the study, i.e., in a group of women between their 2nd and 4th postpartum day. As the factor loading for item 17, A mother who occasionally drinks alcohol should not breastfeed her baby, did not reach the criterion value, the item was not included in further analyses. The reliability of the 16-item scale, measured by Cronbach’s α, was 0.725, which is satisfactory and comparable to that found for the Spanish and Greek versions of the questionnaire, though lower than that obtained in the original study by De la Mora et al. [14,20,26].

The scale correlation analysis showed that all 16 scale items were positively correlated. The correlations ranged between 0.201 and 0.492, indicating that the items of the measure provide an accurate assessment of attitudes towards infant feeding. Regarding item 17 of the IIFSA (A mother who occasionally drinks alcohol should not breastfeed her baby), similar findings were reported by Ho et al. (2011), Nanishi et al. (2014), and Charafeddine et al. (2016) [27,28]. Conversely, Iliadou et al. (2019) showed a good corrected item–total correlation for item 17 in their study in a group of Greek women, explaining the finding by the fact that in the Greek society, occasional alcohol consumption is considered acceptable in the cultural and religious context [26].

In the present study, we also analyzed the mean IIFAS score. Inoue et al. reported a mean total IIFAS score of 54.2 (±4.9) in Japanese mothers [9]. Chen et al. (2013) reported means of 60.0 (±6.3) in Chinese mothers living in Australia and 57.7 (±5.1) in Chinese mothers living in China [8]. In Jordanian mothers, the mean score was 63.5 (±4.67), Spanish—69.76 (±7.75), and Hungarian—66.76 (±9.0) [11,14,29]. The mean scores cited above, as well as the present mean score of 63.12 (±7.34), show that women are consistently found to have a neutral attitude towards breastfeeding. Higher scores, indicating a positive attitude towards breastfeeding, were found in Greek women: 70.0 (±7.6) [26].

A neutral attitude towards breastfeeding does not have to signify a lack of a well-established approach in this area, but it can rather be a sign of tolerance and understanding with regard to differing opinions about infant feeding.

During part one of our study, performed in obstetric wards, we were concerned about respondents providing socially acceptable answers about breastfeeding, but an analysis of feeding attitudes in the same group 2 and 6 months after delivery showed no changes in these attitudes (95% confidence interval).

Furthermore, we analyzed breastfeeding attitudes in relation to socio-demographic factors. There was a weak correlation between the age and the attitude of women towards breastfeeding. Mathew et al. (2019) demonstrated a significantly shorter duration of breastfeeding in mothers aged 15–24 years, which indicates that this group of women requires more education and support in this area [30]. Sarki et al. analyzed the relationship between mothers’ education and duration of breastfeeding and found that those who had completed higher education were more likely to breastfeed and continued breastfeeding for a longer time than those with lower education levels [31]. In our study, women who had completed higher education had a more positive attitude towards breastfeeding, which is likely to be associated with a greater tendency to seek knowledge on the health aspects of infant nutrition.

The present study also demonstrates that women who are married or in a steady relationship have a more positive attitude towards breastfeeding than other women. Masho et al. (2016) showed that unmarried women had greater odds of never breastfeeding and of breastfeeding for 8 weeks or shorter, compared with married women who tended to breastfeed for more than 8 weeks [32].

Data on the association between socio-economic standing and duration of breastfeeding are inconsistent. Bareness et al. (2021) reported that in developing countries, women with the highest socio-economic status tended to discontinue breastfeeding earlier [33]. In turn, Persad et al. found positive attitudes towards breastfeeding in higher-income respondents [34].

One of the priorities of the Global Breastfeeding Collective is to ensure that women receive paid maternity leave and can breastfeed at work. Data on Poland in the report are limited due to insufficient monitoring in the country, but the fully paid maternity leave available to women in the country is viewed favorably [17].

Our study shows that women who are professionally active before the pregnancy and plan to return to work after their maternity leave have a more positive attitude towards breastfeeding than the remaining respondents. Notably, though, Perera et al. (2021) report that returning to work after childbirth is one of the reasons why women discontinue exclusive breastfeeding [35]. Balogun et al. (2015), who also identified return to work as a significant barrier to exclusive breastfeeding, emphasized that short maternity leaves or a lack of facilities for breastfeeding in the workplace were among the reasons [36]. Another potential reason is a lack of spaces for pumping breast milk. Currently in Poland, women can benefit from 20 weeks’ fully paid maternity leave. Upon the return to work, breastfeeding women are entitled to two 30 min breaks included in their working time. The two breaks may be combined, in which case they are still included in working time [37].

Laanterä et al. demonstrated a more positive attitude toward breastfeeding among parents who have at least one child, have completed higher education, and have a high level of knowledge on breastfeeding [38]. Mbada et al. found that multiparity and previous preparation for lactation positively affected attitudes towards breastfeeding [39].

In our study, there was no association between mode of delivery and attitude toward breastfeeding. However, in Shosha et al. (2015), more positive attitudes towards breastfeeding were found in women who delivered vaginally and who gave birth to a healthy baby, and less positive ones in those who gave birth prematurely and whose babies were treated in a neonatal intensive care unit [11].

The decision on the way of feeding is often made before delivery, and breastfeeding is typically seen as a woman’s personal choice [40]. Still, despite their initial declaration regarding plans to breastfeed, many mothers choose formula feeding once they have given birth, which is associated with socio-demographic, health, and psychological factors, as well as difficulties experienced during lactation. A 2014 study performed in Poland demonstrated that 97.0% of mothers breastfed shortly after delivery, but the percentage fell to 43.5% 2 months after delivery, and to 4% 6 months after delivery [24]. On the other hand, Weker et al. (2016) in their study on a representative sample of children (n = 1059) showed that approx. 10% of children aged 13–36 months were still breastfed [41].

The vast majority of the Polish respondents planned to breastfeed their baby exclusively and showed a positive attitude towards this way of feeding, and as emphasized by Guelinckx et al. (2021), even just the intention to breastfeed is positively associated with later breastfeeding behaviors [42].

The present study warrants the conclusion that particular attention and support should be given to younger women, with primary or vocational education, with an unsatisfactory socio-economic standing, those who are professionally inactive, and those who plan on formula or combined feeding already in the first postpartum days. In the new situation for the woman, professional support and lactation counseling may be crucial to the initiation and continuation of breastfeeding and to shaping positive attitudes towards this way of feeding, as emphasized, e.g., by Pérez-Escamilla et al. in their review of studies from 19 countries on the impact of following the “10 steps to successful breastfeeding” [43].

In Poland, the official Perinatal Care Standard requires all mothers to be provided with lactation counseling in the hospital and over the first postpartum weeks. An important provision states that in the hospital, newborns may only be fed with formula on the mother’s explicit request, or on the physician’s orders motivated by health reasons. As part of their health insurance coverage, within the first 2 months after delivery, mothers have access to support from community midwives, tasked with promoting breastfeeding and providing education on and support in lactation. Sadly, lactation counseling beyond 2 months is not covered by the national health insurance. Lactation clinics and lactation consultants operate on a commercial or pro bono basis [44].

In the literature on the subject, authors emphasize the need to develop effective methods of promoting breastfeeding that would help shape the desired attitude towards breastfeeding and improve its social perception. Education and support are also important so that women regain confidence in breastfeeding. Providing knowledge on the benefits of breastfeeding or methods for addressing any difficulties encountered, as well as support and assistance to women who are breastfeeding, should be prioritized to strengthen the role of breastfeeding, increase the number of women who choose breastfeeding, and extend the duration of breastfeeding. Besides healthcare professionals, social campaigns in the mass media and other efforts should promote breastfeeding by focusing on changing attitudes and raising awareness of this way of feeding [31,45]. This is particularly important in the time of the COVID-19 pandemic. The WHO recommends that mothers with suspected or confirmed COVID-19 should be encouraged to initiate or continue to breastfeed. Mothers should be counseled that the benefits of breastfeeding substantially outweigh the potential risks for transmission [46].

An understanding of both positive attitudes and misconceptions and of women’s level of knowledge about infant feeding, and especially breastfeeding, will enable the needs of mothers and their children to be properly addressed. The IIFAS can help in identifying specific misconceptions about breastfeeding prevalent among women. Both knowledge and attitude are variables that can be modified so as to improve breastfeeding practices. One strength of our study lies in the fact that this is the first Polish study to investigate women’s attitudes toward breastfeeding using a standardized instrument, the Iowa Infant Feeding Attitudes Scale (IIFAS), and that the respondents were contacted in person during the first two parts. Validation of this scale will not only enable the practical application of the questionnaire in Poland, but also ensure the comparability of findings with those from other countries and cultures. To make our study even more reliable, we performed it in different regions of Poland.

Despite the differences in the literature regarding the required sample sizes for instrument validation, we chose a sample size of 300–450 as one allowing acceptable pattern compatibility to be observed [47]. The original IIFAS questionnaire comprises 17 items, but since our analysis showed that the factor loading of one item did not reach the criterion value, we excluded this item from further analyses. Thus, the final Polish version of the IIFAS comprises 16 items (Appendix A Table A1). In terms of the limitations of the present study, one notable characteristic is the racially and culturally homogeneous sample. On the other hand, the study pertained to the implementation of the IOWA scale in Poland, whose culture is not highly varied. Therefore, obtaining heterogeneous samples proved to be impossible. To obtain as varied data as possible, the study was conducted in four geographically different areas in Poland, with random sampling. Furthermore, we did not collect information on such variables as social support, breastfeeding education, or any lactation problems experienced. As univariate analysis was used to explore the impact of socio-demographic factors on Polish women’s attitudes towards breastfeeding, further research using in-depth statistical analysis is recommended to explore the value of multivariate analysis on individual factors in relation to the scale. Another aspect of our study is that it was conducted during the COVID 19 pandemic. The results which we obtained showed a neutral attitude towards breastfeeding, as in most European countries where similar research was carried out before the pandemic. However, this topic should be continued, and it requires further research in this direction.

## 5. Conclusions

The Polish version of the IIFAS is a reliable and appropriate measure of women’s attitudes towards infant feeding in Polish settings, with acceptable psychometric properties and construct validity.

The validation of the IIFAS in a Polish setting will enable the investigation of women’s attitudes towards breastfeeding and a comparison of findings with those obtained in other countries.

The scale enables the identification of women who are less likely to breastfeed and highlights any misconceptions about lactation. Understanding attitudes towards infant feeding may prove useful in targeting and evaluating breastfeeding-promoting interventions.

## Figures and Tables

**Table 1:** Participants’ characteristics.

Participants’ Characteristics		*N*	%
Mean age (SD)	29.90 (± 4.95), range 18–43 y/o *		
Residence	Urban—province capital	250	62.3
	Urban—other	68	17.0
	Rural	83	20.7
Education	Primary/vocational	40	10.0
	High school	110	27.4
	College/university	251	62.6
Relationship status	Single	61	15.2
	Married/in a stable relationship	340	84.8
Self-assessed socio-economic status	Very good, good	165	41.1
	Average	228	56.9
	Bad/very bad	8	2.0
Worked before the pregnancy	Yes	329	82.0
	No	72	18.0
Return to work after the end of maternity leave	Yes	323	80.5
	No	78	19.5
Mode of delivery	Vaginal delivery	217	54.1
	Cesarean section	184	45.9
Parity	1	195	48.6
	2	144	35.9
	3 or higher	62	15.5
Way of feeding the previous baby	Breastfeeding	132	64.1
	Formula/combined	74	35.9
Planned way of feeding now	Breastfeeding	324	80.8
	Formula/combined	77	19.2

* y/o—years old.

**Table 2:** Psychometric properties of the Polish version of the IIFAS-Pol * scale.

Items	Positive Attitude towards Breastfeeding	Positive Attitude towards Formula Feeding	Item-Total Correlations	Cronbach’s α If Item Deleted
1. The nutritional benefits of breast milk last only until the baby is weaned from breast milk.		0.501	0.238	0.721
2. Formula feeding is more convenient than breast-feeding.		0.387	0.323	0.712
3. Breast-feeding increases mother–infant bonding.	0.467		0.214	0.722
4. Breast milk is lacking in iron.		0.608	0.201	0.728
5. Formula-fed babies are more likely to be overfed than breast-fed babies.	0.559		0.311	0.713
6. Formula-feeding is the better choice if a mother plans to work outside the home.		0.352	0.274	0.718
7. Mothers who formula-feed miss one of the great joys of motherhood.	0.651		0.398	0.703
8. Women should not breast-feed in public places such as restaurants.		0.452	0.201	0.724
9. Babies fed breast milk are healthier than babies who are fed formula.	0.687		0.377	0.706
10. Breast-fed babies are more likely to be overfed than formula fed babies.		0.501	0.387	0.706
11. Fathers feel left out if a mother breast-feeds.		0.715	0.289	0.715
12. Breast milk is the ideal food for babies.	0.570		0.380	0.708
13. Breast milk is more easily digested than formula.	0.535		0.395	0.705
14. Formula is as healthy for an infant as breast milk.		0.631	0.492	0.694
15. Breast-feeding is more convenient than formula feeding.	0.570		0.410	0.702
16. Breast milk is less expensive than formula.	0.505		0.313	0.714
17. A mother who occasionally drinks alcohol should not breast-feed her baby.	−0.194	−0.184	---	---
**%** of variance explained	19.90	11.28	---	---

* analysis based on data obtained in the 1st stage of the research.

**Table 3:** Women’s attitudes towards breastfeeding in the first 2–4 days postpartum, after 2 months, and after 6 months.

Attitude towards Feeding	1st Stage of Research * M (±SD) (95% CI)	2nd Stage of Research ** M (±SD) (95% CI)	3rd Stage of Research *** M (±SD) (95% CI)	Statistical Analysis	
				F	*p*
Positive attitude towards breastfeeding	32.67 (±4.57) (32.22–33.12)	32.80 (±4.57) (32.37–33.23)	32.04 (±3.82) (31.62–32.60)	2.149	0.117
Positive attitude towards formula feeding	30.45 (±4.57) (30.00–30.90)	30.81 (±4.13) (28.36–31.28)	30.28 (±4.16) (29.76–30.76)	1.105	0.332
Total score	63.12(±7.34) (62.40–63.85)	63.60 (±6.29) (62.90–64.37)	62.29 (±6.52) (61.36–63.17)	2.251	0.106

* 2–4 days after delivery, ** 2 months after delivery, *** 6 months after delivery, F—The One-Way Repeated Measures ANOVA; 95% CI—95% confidence interval.

**Table 4:** Socio-demographic variables and women’s attitudes towards breastfeeding—IIFAS-Pol scores *.

Variables		PolIIFAS		Statistical Analysis	
		M	(±SD)	F/t	*p*
Age	0.108 **				0.031
Residence	Urban—province capital	63.83	7.37	3.227	0.041
	Urban—other	61.60	7.48		
	Rural	62.25	6.96		
Education	Primary/vocational	61.08	6.24	3.541	0.030
	High school	62.25	7.39		
	College/university	63.83	7.41		
Relationship status	Single	59.05	5.89	−4.837	<0.001
	Married/in a stable relationship	63.86	7.35		
Perceived family wealth	Very wealthy/rather wealthy	64.20	7.39	3.481	0.032
	Average	62.46	7.18		
	Rather poor/poor	60.00	8.78		
Worked before the pregnancy	Yes	63.89	7.12	4.594	<0.001
	No	59.61	7.35		
Return to work after the end of maternity leave	Yes	63.97	7.10	4.814	<0.001
	No	59.63	7.33		
Parity	1	63.30	7.28	0.500	0.607
	2	62.66	7.78		
	3 or higher	63.65	6.49		
Mode of delivery	Vaginal delivery	63.62	6.86	1.462	0.145
	Cesarean section	62.54	7.86		
Way of feeding the previous baby	Breastfeeding	64.30	6.87	6.969	<0.001
	Formula/combined	58.17	7.23		
Planned way of feeding now	Breastfeeding	63.95	6.40	2.618	0.010
	Formula/combined	61.18	8.71		

Note: * analysis based on data obtained in the 1st stage of the research, F—single-factor ANOVA, t—*t*-test for independent samples; ** for age, Pearson’s r was used.

## Data Availability

The datasets generated during and/or analyzed during the current study are available from the corresponding author on reasonable request.

## Appendix A

Proszę o wskazanie, w jakim stopniu zgadza się lub nie zgadza się Pani z każdym z poniższych stwierdzeń poprzez zaznaczenie kółkiem cyfry, która najlepiej odzwierciedla Pani opinię, przyjmując, że “1” oznacza zdecydowanie się nie zgadzam, a “5” oznacza zdecydowanie się zgadzam. Może Pani wybrać dowolną liczbę od 1 do 5.

**Table A1:** The Iowa Infant Feeding Attitude Scale—Polish version.

1.	Korzyści z karmienia piersią trwają tylko do momentu odstawienia dziecka od piersi.	1	2	3	4	5
2.	Karmienie mieszanką sztuczną jest wygodniejsze niż karmienie piersią.	1	2	3	4	5
3.	Karmienie piersią wzmacnia więź między matką a dzieckiem.	1	2	3	4	5
4.	W mleku matki brakuje żelaza.	1	2	3	4	5
5.	Niemowlęta karmione mieszanką sztuczną są częściej przekarmiane niż dzieci karmione piersią.	1	2	3	4	5
6.	Karmienie mieszanką sztuczną jest lepszym rozwiązaniem dla matki, która chce wrócić do pracy.	1	2	3	4	5
7.	Matki karmiące mieszanką sztuczną tracą jedną z największych radości macierzyństwa.	1	2	3	4	5
8.	Kobiety nie powinny karmić piersią w miejscach publicznych, takich jak restauracje.	1	2	3	4	5
9.	Niemowlęta karmione piersią są zdrowsze niż dzieci karmione mieszanką sztuczną.	1	2	3	4	5
10.	Niemowlęta karmione piersią są częściej przekarmiane niż dzieci karmione mieszanką sztuczną.	1	2	3	4	5
11.	Ojcowie czują się pominięci, jeśli matka karmi piersią.	1	2	3	4	5
12.	Mleko matki jest idealnym pożywieniem dla niemowląt.	1	2	3	4	5
13.	Mleko matki jest bardziej lekkostrawne niż mieszanka sztuczna.	1	2	3	4	5
14.	Mieszanka sztuczna jest tak samo zdrowa dla niemowlęcia jak mleko matki.	1	2	3	4	5
15.	Karmienie piersią jest wygodniejsze niż karmienie mieszanką sztuczną.	1	2	3	4	5
16.	Mleko matki jest tańsze niż mieszanka sztuczna.	1	2	3	4	5

1: Zdecydowanie się nie zgadzam. 2. Nie zgadzam się. 3. Nie mam zdania. 4. Zgadzam się. 5. Zdecydowanie się zgadzam.
